# Tenosynovial Giant Cell Tumors: A Musculoskeletal Case Series

**DOI:** 10.7759/cureus.92200

**Published:** 2025-09-13

**Authors:** Franco C Yip, Yung Kong Wong, Wing Ho Chong, On Chee Li, Kai Yan Kwok

**Affiliations:** 1 Department of Radiology and Nuclear Medicine, Tuen Mun Hospital, Tuen Mun, HKG

**Keywords:** joint disease, magnetic resonance imaging, pigmented villonodular synovitis, synovitis, tenosynovial giant cell tumor

## Abstract

Tenosynovial giant cell tumors (TSGCT) are uncommon soft tissue neoplasms that can become debilitating if not diagnosed and treated promptly, particularly in advanced stages. Because of their nonspecific clinical manifestations, they are often misdiagnosed or overlooked. This case series highlights the varied clinical presentations and characteristic imaging findings of patients with TSGCT. By addressing key diagnostic challenges and pitfalls, the study aims to improve awareness and enhance diagnostic accuracy, thereby supporting timely and effective management in clinical practice.

## Introduction

Tenosynovial giant cell tumors (TSGCT), previously termed pigmented villonodular synovitis, are benign hypertrophic processes of the synovium characterized by villous, nodular, and villonodular proliferation with associated hemosiderin deposition [1]. This uncommon disease originates from the synovium of joints, bursae, or tendon sheaths and is classified into localized and diffuse types, as well as intra-articular or extra-articular forms [1-4]. The localized type (L-TSGCT) is the most common, typically extra-articular, and often affects the tendon sheaths of the hands and feet, whereas the intra-articular form most frequently involves the knees [1-5]. The diffuse type (D-TSGCT) arises predominantly in intra-articular spaces and most commonly affects the knees, hips, ankles, shoulders, and elbows [1,4-6].

Clinical manifestations of TSGCT are often nonspecific and variable, which may result in delayed or missed diagnoses. Preliminary imaging techniques such as radiography, USG, or CT provide limited diagnostic information, and the presence of arthropathic changes can further complicate evaluation.

MRI is regarded as the gold standard for diagnosing TSGCT, as it allows precise lesion localization and detailed assessment of adjacent anatomical structures [2,5]. This level of accuracy supports treatment planning and helps reduce the risk of incomplete tumor resection and disease recurrence. Postoperative MRI is also valuable for monitoring recurrence [1,2].

This case series presents three Chinese patients aged 21 to 65 years and aims to increase awareness of TSGCT as a differential diagnosis in patients presenting with nonspecific joint pain, with or without periarticular masses. Various imaging modalities are discussed, with MRI emphasized as the preferred tool due to its superior ability to characterize synovial proliferation, hemosiderin deposition, and joint involvement. Key imaging features across modalities are highlighted to promote earlier and more accurate diagnosis.

## Case presentation

Case 1

**Figure 1:**
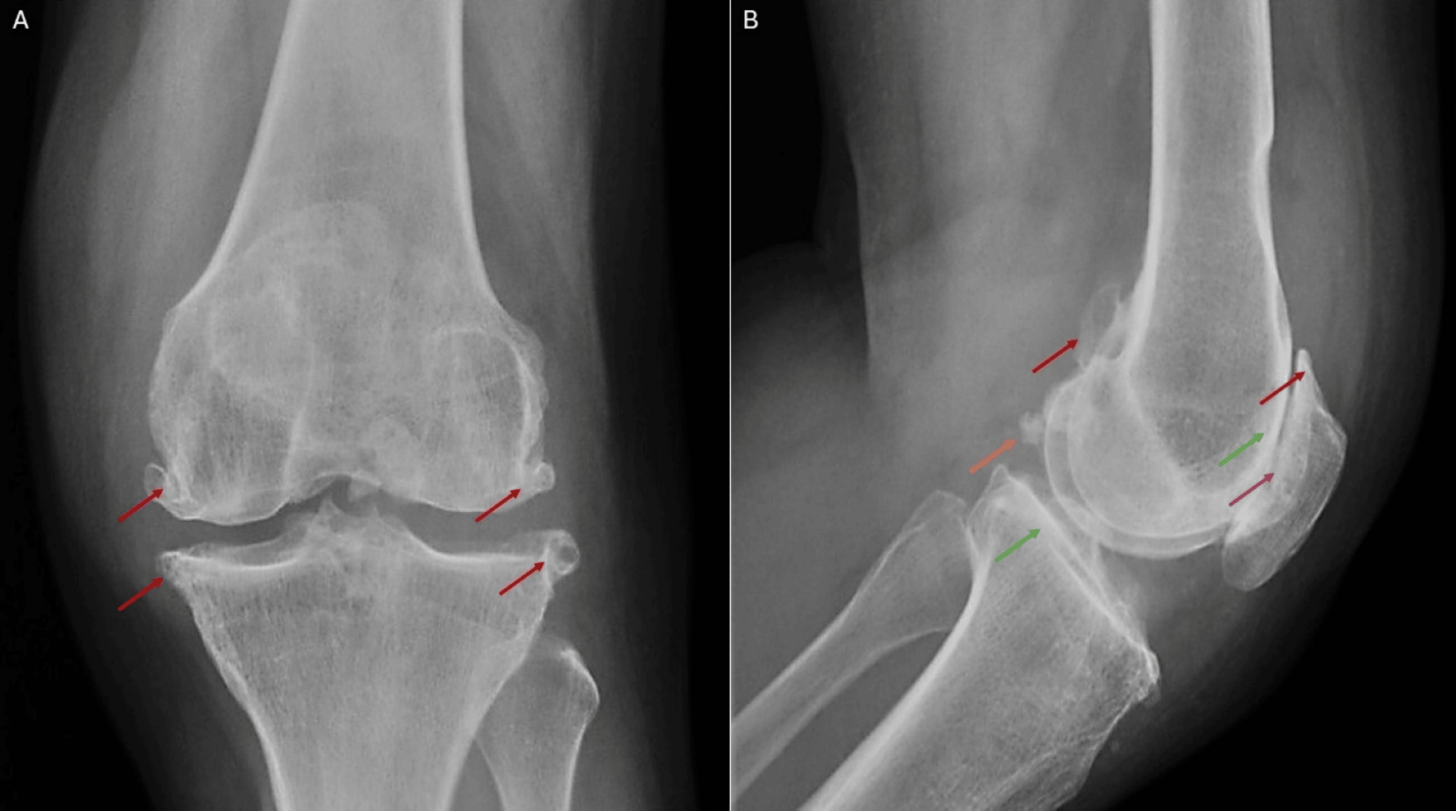
Radiographs of the left knee demonstrate subchondral sclerosis (green arrows), joint space narrowing in the patellofemoral compartment (purple arrow), osteophytes (red arrows), and intra-articular bodies (orange arrow). (A) Frontal knee radiograph. (B) Lateral knee radiograph.

A 65-year-old female with chronic left knee pain was admitted to the Department of Orthopedics for evaluation of pain and swelling in the left knee. There was no history of trauma, and she was able to walk unaided. Physical examination revealed joint effusion, crepitus, and tenderness along the medial joint line, with a full active range of motion. Radiographs demonstrated subchondral sclerosis, joint space narrowing in the patellofemoral compartment, osteophytes, and intra-articular bodies (Figure 1A, 1B). Aspiration of the knee joint yielded hemarthrosis without fat globules, crystals, or bacterial growth on culture. The patient was initially managed conservatively with analgesics, discharged, and scheduled for follow-up in the orthopedic clinic.

**Figure 2:**
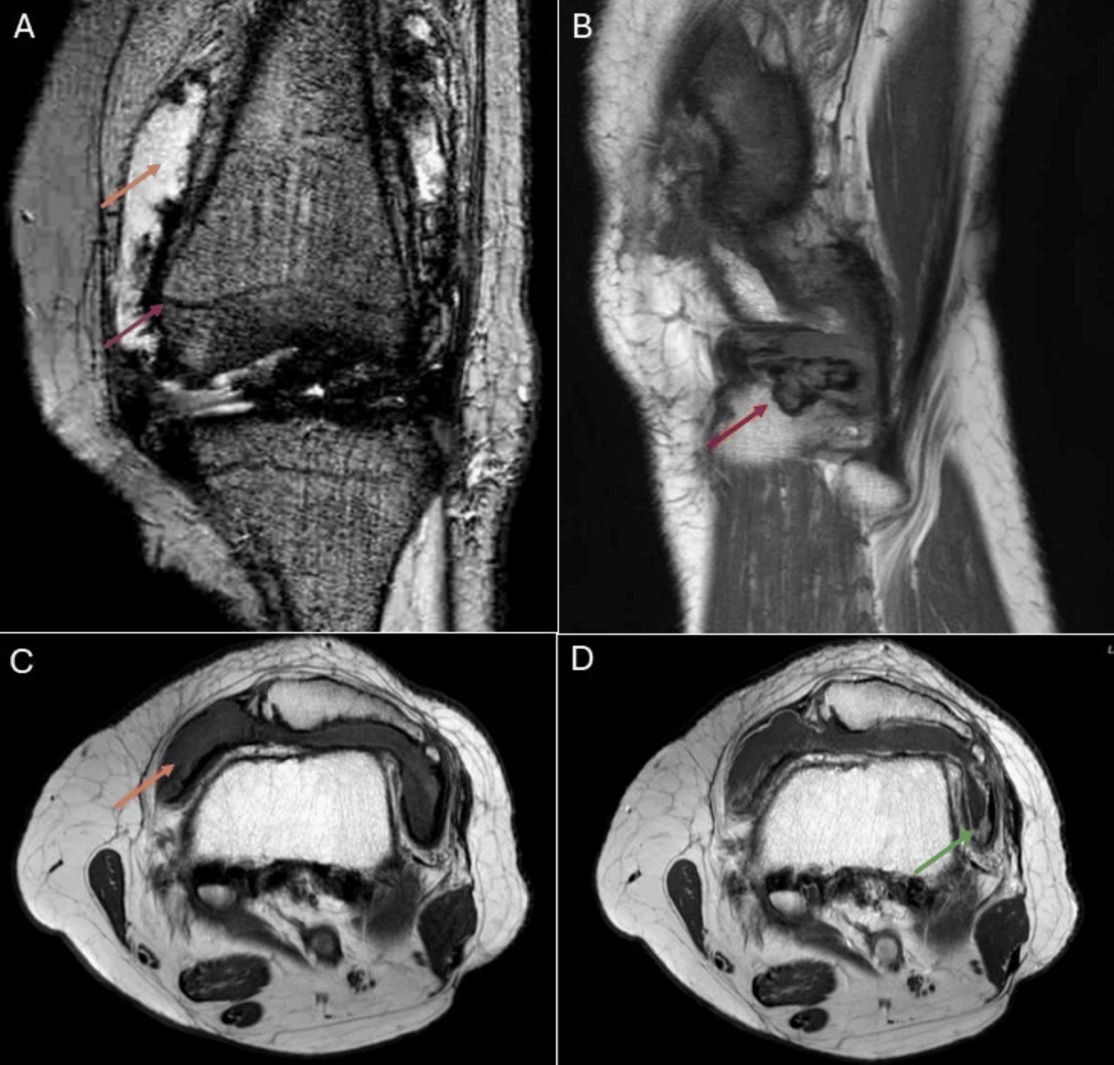
MRI of the left knee shows diffuse enhancing synovial thickening (green arrow). The thickened synovium appears markedly hypointense on gradient echo sequences (purple arrow), suggestive of diffuse hemosiderin deposition. This synovial thickening also caused pressure erosion along the lateral margin of the lateral tibial plateau (red arrow). Additionally, moderate joint effusion is noted in the left knee (orange arrows). (A) Gradient echo sequence, coronal projection. (B) T1-weighted sequence, sagittal projection. (C) T1-weighted sequence, axial projection. (D) T1-weighted sequence with contrast, axial projection.

A gadolinium-enhanced MRI of the left knee was performed four months later (Figure 2A-2D). It demonstrated diffuse enhancing synovial thickening. The thickened synovium appeared markedly hypointense on gradient echo sequences, suggestive of diffuse hemosiderin deposition. The synovial thickening also caused pressure erosion along the lateral margin of the lateral tibial plateau. A moderate joint effusion was also present in the left knee.

The patient discussed treatment options with the orthopedic surgeons and opted for arthroscopic synovectomy. Given the diagnosis of TSGCT with concomitant severe osteoarthritis of the knee, synovectomy was selected as a less invasive procedure aimed at controlling synovitis and hemosiderin deposition while preserving joint integrity. Intraoperative findings included blood-stained effusion and diffuse synovial thickening with extensive hemosiderin deposition. Synovitis was observed in the patellofemoral joint space, intercondylar notch, and both medial and lateral compartments. Histopathological analysis confirmed the diagnosis of TSGCT. Following surgery, the patient underwent physiotherapy and regained the ability to walk unaided, with no joint swelling and only minimal residual pain. She was then lost to follow-up.

Case 2

**Figure 3:**
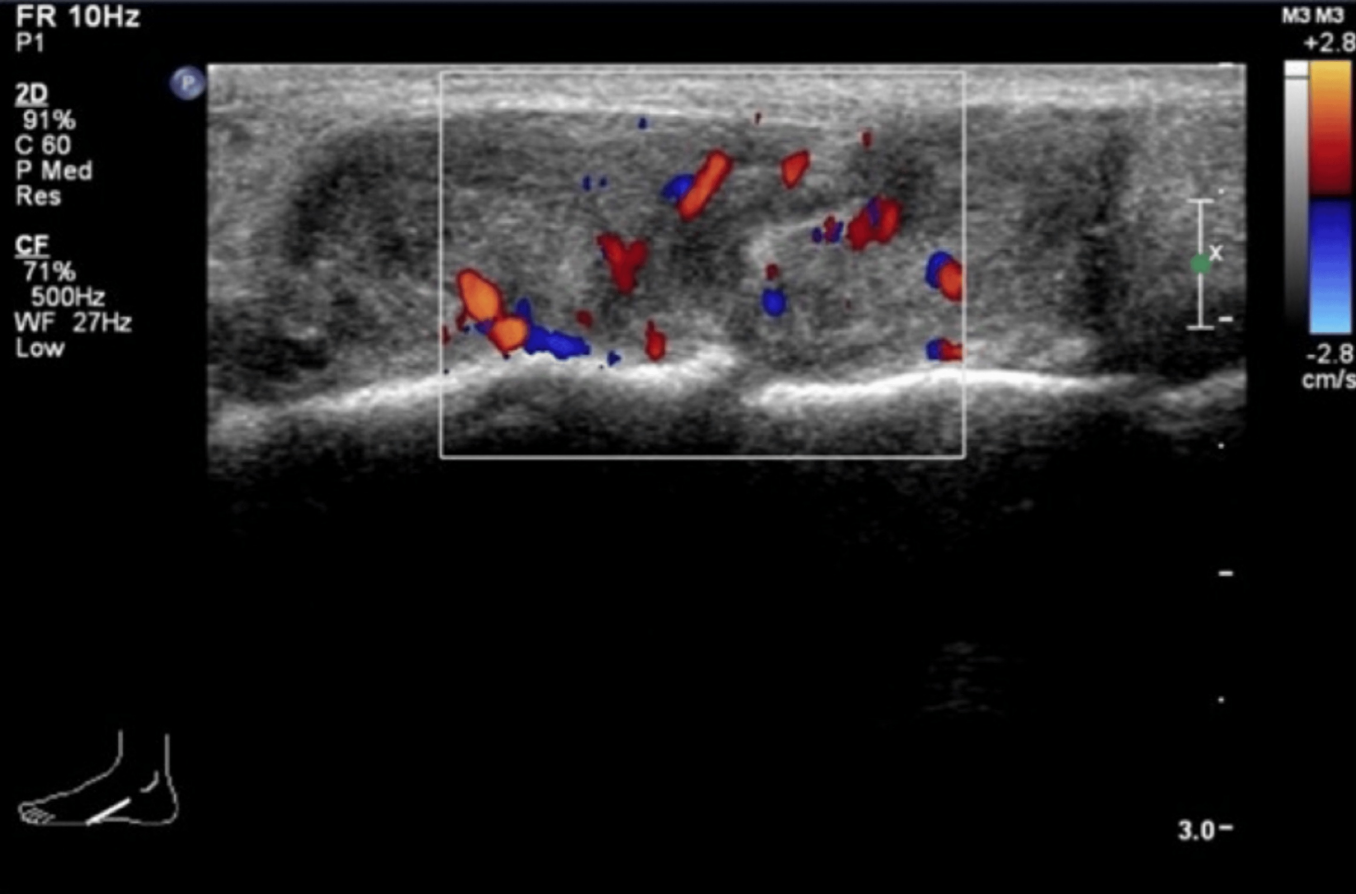
Ultrasound examination of the left foot reveals a vascular, isoechoic lesion with a lobulated contour on the dorsum, shown in the longitudinal projection.

A 51-year-old female presented with a slow-growing mass on the dorsum of her left midfoot, present for six months without any history of trauma. Physical examination revealed an immobile mass on the left midfoot that was not attached to the overlying skin. There were no associated skin changes or signs of transillumination. Ultrasound examination demonstrated a vascular, isoechoic lesion with a lobulated contour on the dorsum of the left foot (Figure 3).

**Figure 4:**
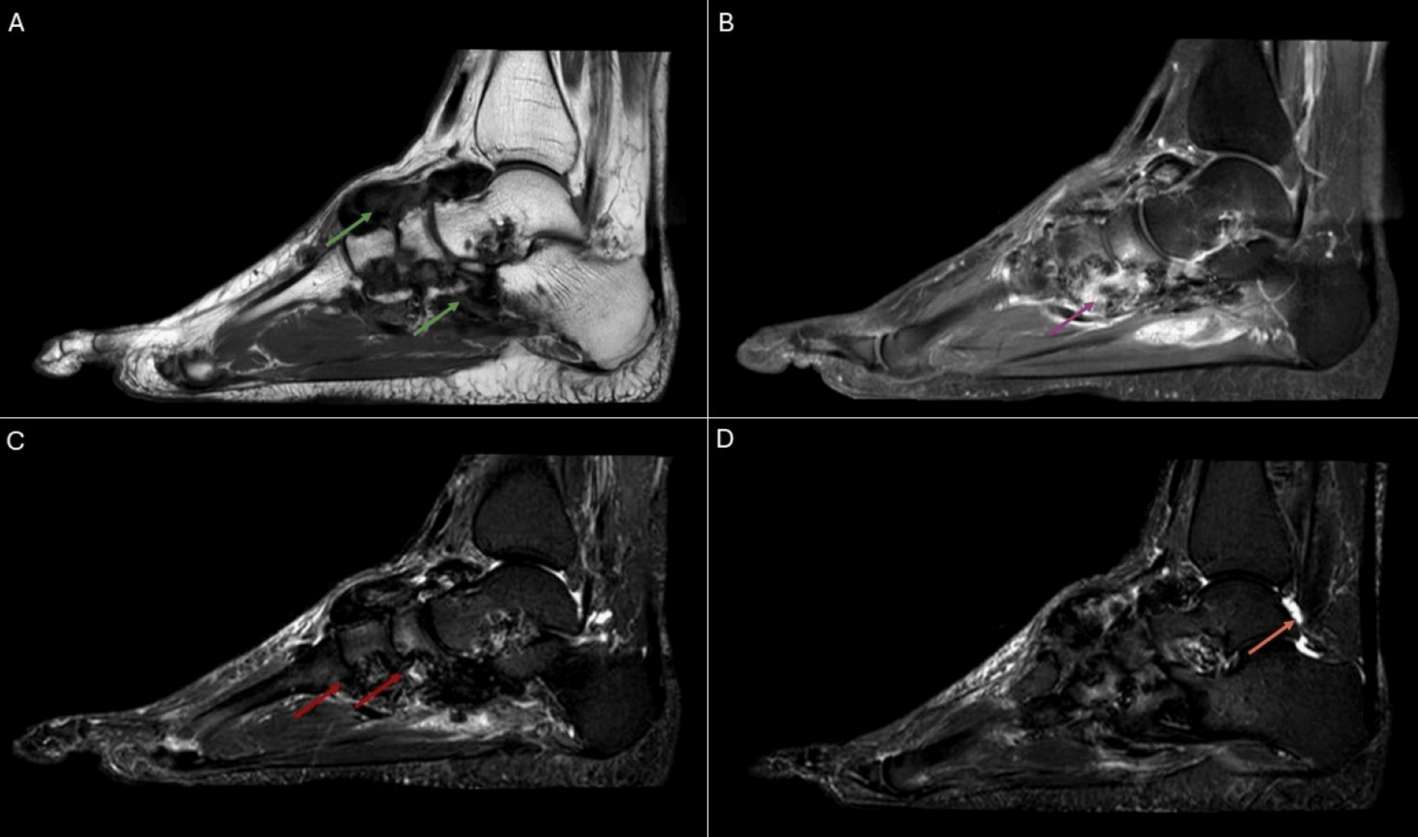
MRI of the left foot shows diffuse synovial thickening over the dorsum with extension toward the plantar region (green arrows). The lesion demonstrates heterogeneous hypointensity on both T1-weighted and T2-weighted sequences, with areas of intense contrast enhancement (purple arrow). Multiple periarticular erosions are present in the intertarsal and tarsometatarsal joints (red arrows). A small effusion is also noted in the left talocrural joint (orange arrow). (A) T1-weighted sequence, sagittal projection. (B) T1-weighted sequence with contrast and fat saturation, sagittal projection. (C, D) T2-weighted sequence with fat saturation, sagittal projection.

Six months later, a gadolinium-enhanced MRI was performed (Figure 4A-4D), which revealed diffuse synovial thickening over the dorsum with extension toward the plantar region. The lesion demonstrated heterogeneous hypointensity on both T1-weighted and T2-weighted sequences, with areas of intense contrast enhancement. Multiple periarticular erosions were observed in the intertarsal and tarsometatarsal joints. A small amount of effusion was also present in the left talocrural joint.

After thorough discussion and in consideration of the highly suggestive MRI findings demonstrating characteristic features of TSGCT, the patient and orthopedic team jointly decided to proceed directly to surgery without a prior biopsy. During surgery, extensive synovial thickening was observed over the dorsum of the foot, extending toward the plantar aspect. Histopathological analysis confirmed the diagnosis of TSGCT. Following the operation, the patient underwent physiotherapy and successfully regained the ability to walk unaided, with no detectable mass or pain.

**Figure 5:**
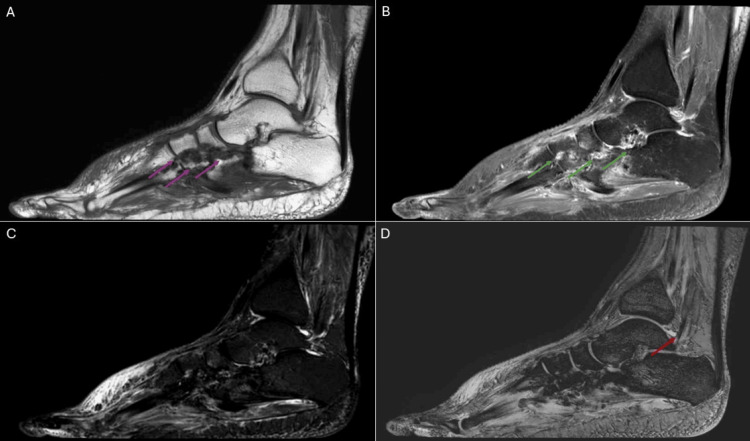
MRI of the left foot demonstrates a reduction in the volume of the index lesion, involving both its dorsal and plantar components. Surrounding enhancement (green arrows) and blooming artifacts are observed, which may represent postoperative inflammatory changes with residual blood products or underlying residual disease. Residual deformities remain at the intertarsal and tarsometatarsal joints, secondary to pressure erosion caused by the disease (purple arrows). A small effusion persists in the left talocrural joint (red arrow). (A) T1-weighted sequence, sagittal projection. (B) T1-weighted sequence with contrast and fat saturation, sagittal projection. (C) T2-weighted sequence with fat saturation, sagittal projection. (D) T2-weighted sequence, sagittal projection.

A postoperative gadolinium-enhanced MRI performed one month later (Figure 5A-5D) demonstrated a reduction in the volume of the index lesion, involving both its dorsal and plantar components. Surrounding enhancement and blooming artifacts were observed, which could represent postoperative inflammatory changes with residual blood products or underlying residual disease. Residual deformities remained at the intertarsal and tarsometatarsal joints, secondary to pressure erosion caused by the disease. A small amount of effusion persisted in the left talocrural joint.

Afterward, the patient was lost to follow-up, and no subsequent information was available from the medical records.

Case 3

A 21-year-old male presented with a slow-growing mass on the posteromedial aspect of his right ankle, which had been present for one month. Notably, he had sustained a right ankle sprain one month prior to presentation. Physical examination revealed a tender, non-pulsatile mass on the posterior aspect of the right ankle. The Tinel sign was negative, and the mass was not attached to the overlying skin.

**Figure 6:**
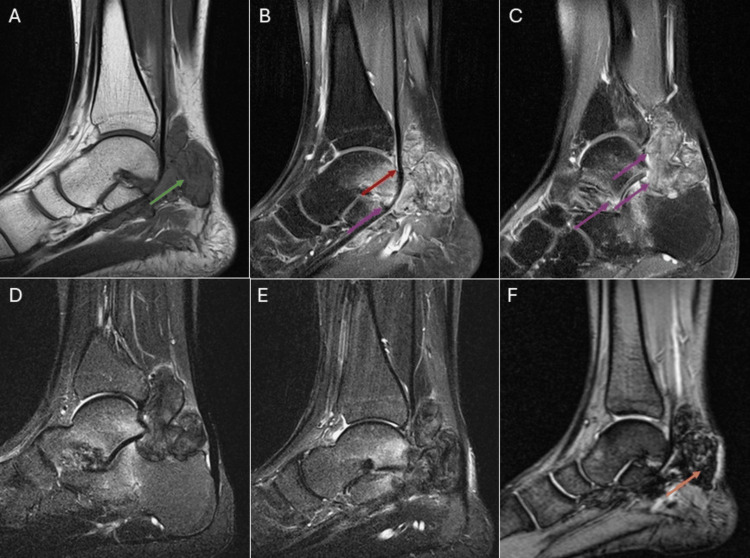
MRI of the right ankle revealed a lobulated mass at the expected location of Kager’s fat pad (green arrow). The mass involved the talocrural and posterior subtalar joints and extended into the sinus tarsi (purple arrows). It encased the flexor hallucis longus tendon (red arrow), extending superiorly around the talocrural joint and inferiorly near the talonavicular joint. The lesion demonstrated intermediate signal intensity on T1-weighted sequences, low signal intensity on T2-weighted sequences, blooming artifacts (orange arrow), and heterogeneous contrast enhancement. (A) T1-weighted sequence, sagittal projection. (B, C) T1-weighted sequence with contrast and fat saturation, sagittal projection. (D, E) T2-weighted sequence with fat saturation, sagittal projection. (F) Gradient echo sequence, sagittal projection.

One month later, a gadolinium-enhanced MRI was performed (Figure 6A-6F). Imaging revealed a lobulated mass in the expected location of Kager’s fat pad. The mass involved the talocrural and posterior subtalar joints and extended into the sinus tarsi. It encased the flexor hallucis longus tendon, extending superiorly around the talocrural joint and inferiorly toward the talonavicular joint. The mass demonstrated intermediate T1-weighted and low T2-weighted signal intensities, with blooming artifacts and heterogeneous contrast enhancement.

**Figure 7:**
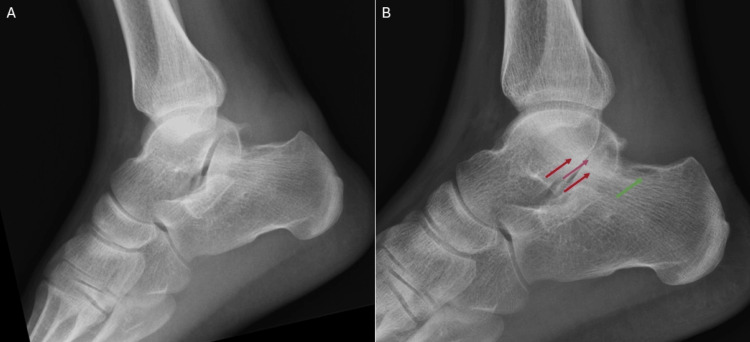
Serial radiographs of the right ankle demonstrated progressive bone remodeling at the posterosuperior aspect of the calcaneus (green arrow), accompanied by degenerative changes in the posterior subtalar joint. These changes included complete loss of joint space (purple arrow) and subchondral sclerosis (red arrows). (A) Initial lateral ankle radiograph. (B) Lateral ankle radiograph at six-month follow-up.

Serial radiographs of the patient’s right ankle (Figure 7A, 7B), obtained six months apart, demonstrated progressive bone remodeling at the posterosuperior aspect of the calcaneus, accompanied by degenerative changes in the posterior subtalar joint. These changes included complete loss of joint space and subchondral sclerosis.

After thorough discussion and in consideration of the highly suggestive MRI findings demonstrating characteristic features of TSGCT, the patient and orthopedic team jointly decided to proceed directly to surgery without a prior biopsy. Intraoperative findings revealed a brown-pigmented nodular mass involving the talocrural and posterior subtalar joints, with extension into the sinus tarsi. Histopathological analysis confirmed the diagnosis of TSGCT. Following surgery, the patient declined physical, medical, and radiation therapies but agreed to undergo interval MRI surveillance, with the next scan pending.

## Discussion

Radiography

The radiographic appearances of TSGCT vary significantly depending on subtype and joint involvement. L-TSGCT typically presents as a well-defined soft-tissue mass, with osseous abnormalities reported in 15-25% of cases [1]. These often include extrinsic bone erosion with well-defined sclerotic margins. In contrast, D-TSGCT may appear normal in up to 21% of cases [1]. When radiological findings are present, D-TSGCT commonly demonstrates joint effusion, soft tissue swelling, and extrinsic bone erosions without soft tissue calcifications. The prevalence of erosive changes depends on the joint involved, with larger joints such as the knees being less prone to bone erosion because of their greater capacity for decompression [2].

CT

CT scans are particularly effective in detecting bone erosions. They can also demonstrate synovial thickening associated with diffuse intra-articular TSGCT. The attenuation of synovial tissue may appear slightly hyperdense compared with adjacent muscles, which can help differentiate TSGCT from other soft tissue masses [1]. In L-TSGCT, CT typically reveals a nonspecific, well-defined soft-tissue mass with muscle-like attenuation. However, CT is less effective than MRI in evaluating the full extent of lesions due to its lower soft tissue resolution, which complicates assessment of surrounding tissue infiltration.

USG

Intra-articular TSGCT, particularly the diffuse form, exhibits characteristic sonographic features, including joint effusion and heterogeneous echogenic masses. The synovial membrane is usually thickened and hypoechoic, with nodular and villous projections reflecting synovial proliferation. Doppler imaging reveals increased vascularity, supporting the diagnosis by indicating active neovascularization associated with tumor growth [1,4,7]. When lesions are visualized, USG can also be useful for guiding biopsies.

MRI

MRI is the preferred modality for diagnosing TSGCT because of its ability to characterize soft tissue tumors and define their extent [1,5,6]. TSGCT typically demonstrates low to intermediate signal intensity on T1-weighted and T2-weighted sequences, with hemosiderin deposition producing blooming artifacts on gradient echo sequences. Contrast enhancement is usually present and often heterogeneous [1,4,5].

In D-TSGCT, MRI often shows extensive synovial membrane involvement with infiltrative margins [1,2,6]. Additional MRI features may include bone erosions, subchondral cysts, intralesional septations, bone marrow edema, and articular cartilage defects [1]. By contrast, L-TSGCT usually appears as a well-circumscribed soft-tissue mass.

Postoperatively, MRI plays a crucial role in surveillance. Local recurrence is defined as the appearance of new disease following synovectomy or the progression of residual disease on follow-up MRI. Within the first six months, diffuse synovial thickening may be equivocal for residual disease due to reactive synovitis. However, disease recurrence should be suspected when there is progressive, enhancing, solid, and nodular synovial thickening [2].

MRI findings following colony-stimulating factor 1 receptor (CSF1R) inhibitor therapy include decreased signal intensity, reduced capsular distension, and increased hemosiderin deposition. These changes are consistent with a positive treatment response in patients receiving CSF1R inhibitors [2].

Diagnosis and treatment

When diagnosing TSGCT with imaging, it is important to avoid several pitfalls to ensure accurate diagnosis and management. A major challenge is the occurrence of TSGCT in atypical locations, which can complicate recognition [3]. L-TSGCT is most often found extra-articularly, commonly affecting the tendon sheaths of the hands and feet [1,2,5]. In contrast, D-TSGCT typically originates intra-articularly, most frequently involving the knees, hips, ankles, shoulders, and elbows [1,3,5]. Despite these typical patterns, clinicians should consider TSGCT in patients with compatible imaging findings, even in less common locations.

Familiarity with characteristic imaging features is essential. These tumors usually show low to intermediate signal intensity on T1-weighted and T2-weighted MRI due to hemosiderin deposition. However, other conditions, such as tophaceous gout, chronic rheumatoid arthritis, and synovial chondromatosis, can mimic TSGCT, making differential diagnosis challenging. MRI aids in distinguishing these entities, but clinical correlation and biopsy may be necessary for definitive diagnosis.

Differentiating between L-TSGCT and D-TSGCT also has important clinical implications. Surgery remains the mainstay of treatment for both types, but achieving complete resection is more challenging in D-TSGCT due to its diffuse nature [1,2,6]. In such cases, medical therapies, including tumor necrosis factor-alpha inhibitors and CSF1R inhibitors, may be considered, especially when surgery is not feasible [6]. Radiation therapy can also serve as an adjunct to surgery, particularly in cases of incomplete resection [1,2,4,6,7].

## Conclusions

Advanced presentations of TSGCT are common due to nonspecific symptoms, often leading to delayed diagnosis. MRI is the preferred imaging modality for evaluating TSGCT because of its ability to characterize soft tissue tumors and identify hemosiderin deposition. Radiography and CT are valuable for detecting bone involvement, while ultrasound is helpful in assessing soft tissue masses and guiding biopsies. Clinicians must remain vigilant to avoid misdiagnosis. This case series emphasizes the importance of heightened clinical awareness of TSGCT.
